# Association between CYP3A4/CYP3A5 genetic polymorphisms and treatment outcomes of atorvastatin worldwide: is there enough research on the Egyptian population?

**DOI:** 10.1186/s40001-023-01038-1

**Published:** 2023-09-27

**Authors:** Mohammed G. Maslub, Mahasen A. Radwan, Nur Aizati Athirah Daud, Abubakar Sha’aban

**Affiliations:** 1 Pharmacy Practice/Clinical Pharmacy Department, Faculty of Pharmacy, Egyptian Russian University, Cairo-Suez Road, Badr City, Cairo, 11829 Egypt; 2 School of Pharmaceutical Sciences, Universiti Sains Malaysia, 11800 USM Pulau Pinang, Malaysia; 3 Division of Population Medicine, Cardiff University, Cardiff, CF14 4YS Wales UK

**Keywords:** Cytochromes P450, CYP3A4, CYP3A5, Polymorphism, Atorvastatin, Adverse effect, Egypt

## Abstract

**Introduction:**

Atorvastatin is regarded as the most frequently prescribed statin worldwide for dyslipidemia. However, clinical response and risk of adverse effects to statin therapy are associated with genetic variations. Numerous research linked statins pharmacokinetics (PK) variations to genetic polymorphisms in cytochromes P450 (CYPs) metabolic enzymes.

**Objective:**

This article reviews the association between CYP3A4/5 genetic variations and response to atorvastatin therapy globally, which includes atorvastatin PK, and the risk for adverse reactions, with a hint to the Egyptians.

**Methods:**

Up to March 30, 2022, electronic medical databases like PubMed, Web of Science, MEDLINE, and Egyptian Knowledge Bank (EKB) were searched. All articles that highlighted the relationship between CYP3A4/5 genetic polymorphisms and atorvastatin efficacy/safety profile were included in this review.

**Results:**

Initially, 492 articles were retrieved after an exhaustive search. There were 24 articles included according to the inclusion criteria. Findings of association studies of CYP3A4/5 genetic polymorphisms with response to atorvastatin varied among different ethnicities. CYP3A4*1B was associated with better therapeutic outcomes after atorvastatin therapy in Chileans and vice versa in Americans. Caucasians with myalgia while using atorvastatin were at significant risk of suffering severe muscle damage if they were carriers of CYP3A5*3/*3. As far as we can report for the Egyptian population, the impact of CYP3A4/5 genetic variations on the response to atorvastatin therapy was understudied.

**Conclusion:**

More pharmacogenetic studies amongst diverse populations worldwide, like the Egyptian population, are necessary to detect further atorvastatin-gene interactions.

**Graphical Abstract:**

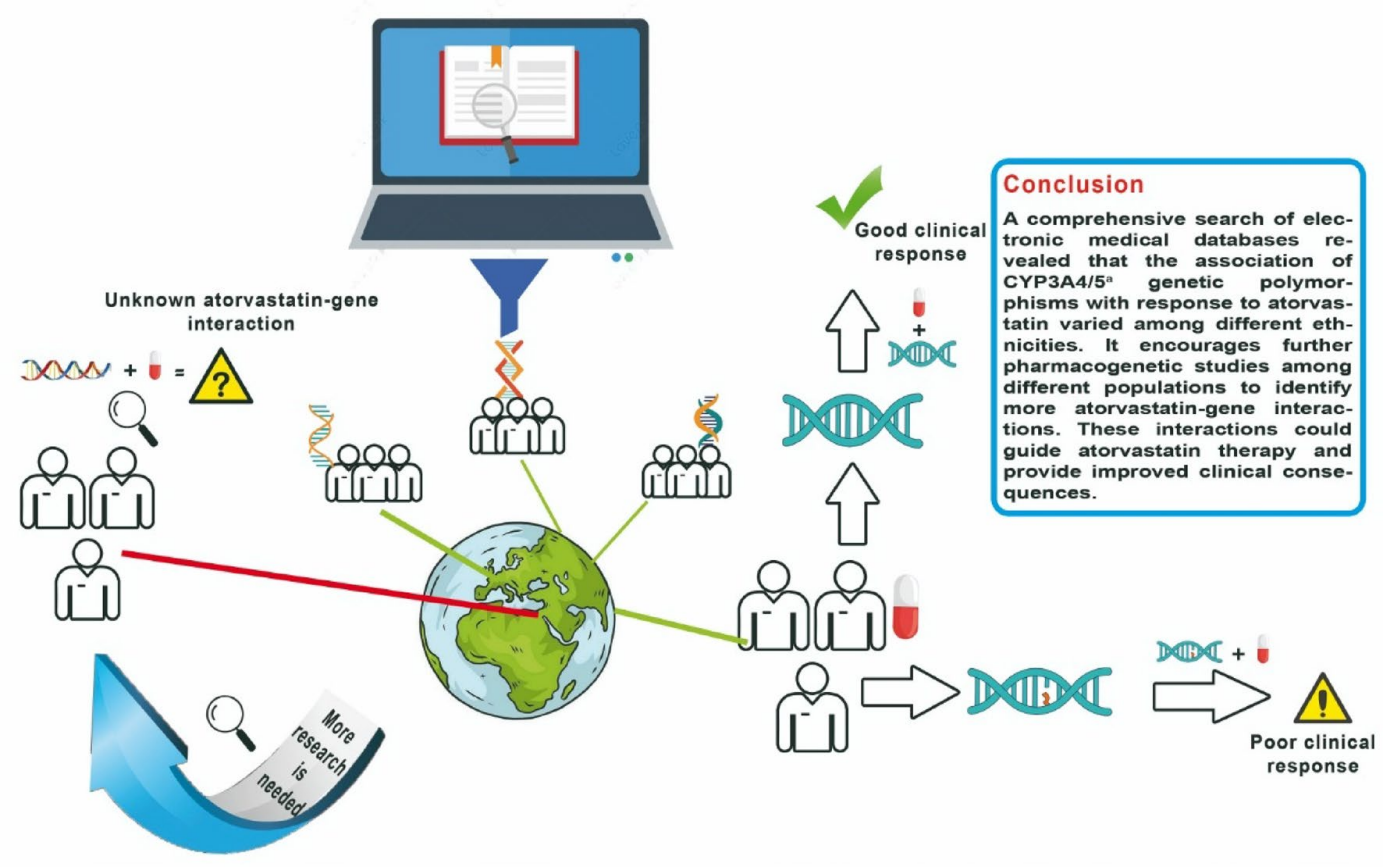

## Keypoints

- CYP3A4/CYP3A5 genetic polymorphisms could influence the efficacy and safety of atorvastatin.
- Numerous research results linking CYP3A4/5 genetic polymorphisms to atorvastatin response varied by ethnicity.
- CYP3A4/5 genetic polymorphisms' role in atorvastatin response in the Egyptian population has received little attention.

## Introduction

*Pharmacogenetics* is a practical approach that predicts both sub-therapeutic responses to pharmacologic treatments and increased risks of adverse drug reactions [1]. Natural genetic variations in human genes result in different responses to drug treatments [2]. Pharmacogenetics is essential in assessing the influence of genetic mutations on responses to pharmacologic therapies [3]. CYPs metabolize a great deal of clinically used medications [4]. Any noticeable change in the levels of the metabolites could occur, in part, due to genetic polymorphisms of these vital enzymes, resulting in changes in the therapeutic outcomes of drugs [5, 6]. The metabolism of drugs is considered an essential process that determines responses to medications, adverse effects, and pharmacokinetics [6]. The strategies of precise medicine will necessitate screening genotypes and phenotypes [7]. Several investigations have been conducted to study the influence of CYPs' genetic variations on the pharmacokinetics of drugs [6]. In the same context, having CYPs polymorphisms can be considered a risk factor for developing myopathy and hepatic injury due to statin therapy [8]. CYPs' genetic variations result in differences in medication responses among people from different ethnicities [9–11].

Differences in the pharmacokinetics of statins were related to genetic polymorphisms in metabolic enzymes such as CYP3A4 and CYP3A5 [12]. Atorvastatin is considered one of the most recommended medications and the utmost extensively prescribed statin worldwide [13]. It is, however, associated with several adverse reactions, such as nausea, nasopharyngitis, insomnia, urinary tract infections, elevation in hepatic enzymes, diarrhea, dyspepsia, myalgia, and arthralgia [14]. Statin-related muscle symptoms are the frequently reported statin-induced reactions, whereas liver toxicities and central nervous system manifestations are less common. Statin-induced adverse reactions lead to noticeable morbidities, more costs, and non-adherence or discontinuation of statin therapy [15, 16].

In Egypt, 46% of overall deaths are related to cardiovascular diseases (CVD) [17, 18]. Dyslipidemia increases the risk of CVD. Several studies showed that high blood cholesterol had been found in 37% of Egyptians [18–20]. Hydroxymethyl glutaryl-CoA (HMG-CoA) reductase inhibitors (statins) are the primary pharmacotherapy for dyslipidemia [21]. *Statin monotherapy* is the lipid-lowering pharmacologic treatment used among Egyptians, with atorvastatin as the most commonly prescribed statin in Egypt [22]. Responses to statins show apparent interpersonal deviations [21]. These response variations develop a significant clinical problem [5, 6].

Results of association studies of either CYP3A4 or CYP3A5 genetic polymorphisms with atorvastatin-induced adverse reactions or atorvastatin efficacy were controversial or inconsistent [23]. Accordingly, this article aims to review the association between CYP3A4/5 genetic variations with response to atorvastatin pharmacological treatment in different ethnic groups, focusing on the Egyptian population.

## Materials and methods

### Study eligibility

This review includes journal articles that are full-text, peer-reviewed, and limited to the English language. The selected articles represented clinical trials, retrospective or prospective observational studies, review articles, or in vitro studies. The nominated articles must include the association between CYP3A4/CYP3A5 polymorphisms and atorvastatin response/safety profile.

### Search strategy

An electronic search was conducted up to March 30, 2022. The search involved electronic medical databases like Web of Science (Clarivate Analytics), PubMed, MEDLINE (Clarivate Analytics), and the appreciable electronic library "Egyptian Knowledge Bank (EKB)," permitting significant search resources absolutely for Egyptians. The following 5 phases queries were used: ((CYP3A5) AND (polymorphism)) AND (atorvastatin) for phase 1, ((CYP3A4) AND (polymorphism)) AND (atorvastatin) for phase 2, (((CYP3A5) AND (polymorphism)) AND (atorvastatin)) AND (adverse effect) for phase 3, (((CYP3A4) AND (polymorphism)) AND (atorvastatin)) AND (adverse effect) for phase 4, and ((Cytochromes P450) AND (atorvastatin)) AND (Egypt) for phase 5.

### Selection of articles

Screening for eligibility was accomplished through three stages: In stage one, the titles were evaluated for relevance. In stage two, abstracts were screened for being eligible. Finally, in stage 3, full-text articles of selected abstracts were assessed (methodology and results) for their eligibility to be included in this review. Exclusion criteria included non-English language literature, duplicate articles, irrelevant methodology to the objectives, and books.

### Data extraction

Data were extracted by (MGM) who separately appraised all the designated articles to extract the relevant ones for this review. One researcher (MGM) accomplished the inclusion process, and in case of uncertainty about article inclusion, a second researcher (MAR) was consulted.

## Results

### Search results

**Fig. 1:**
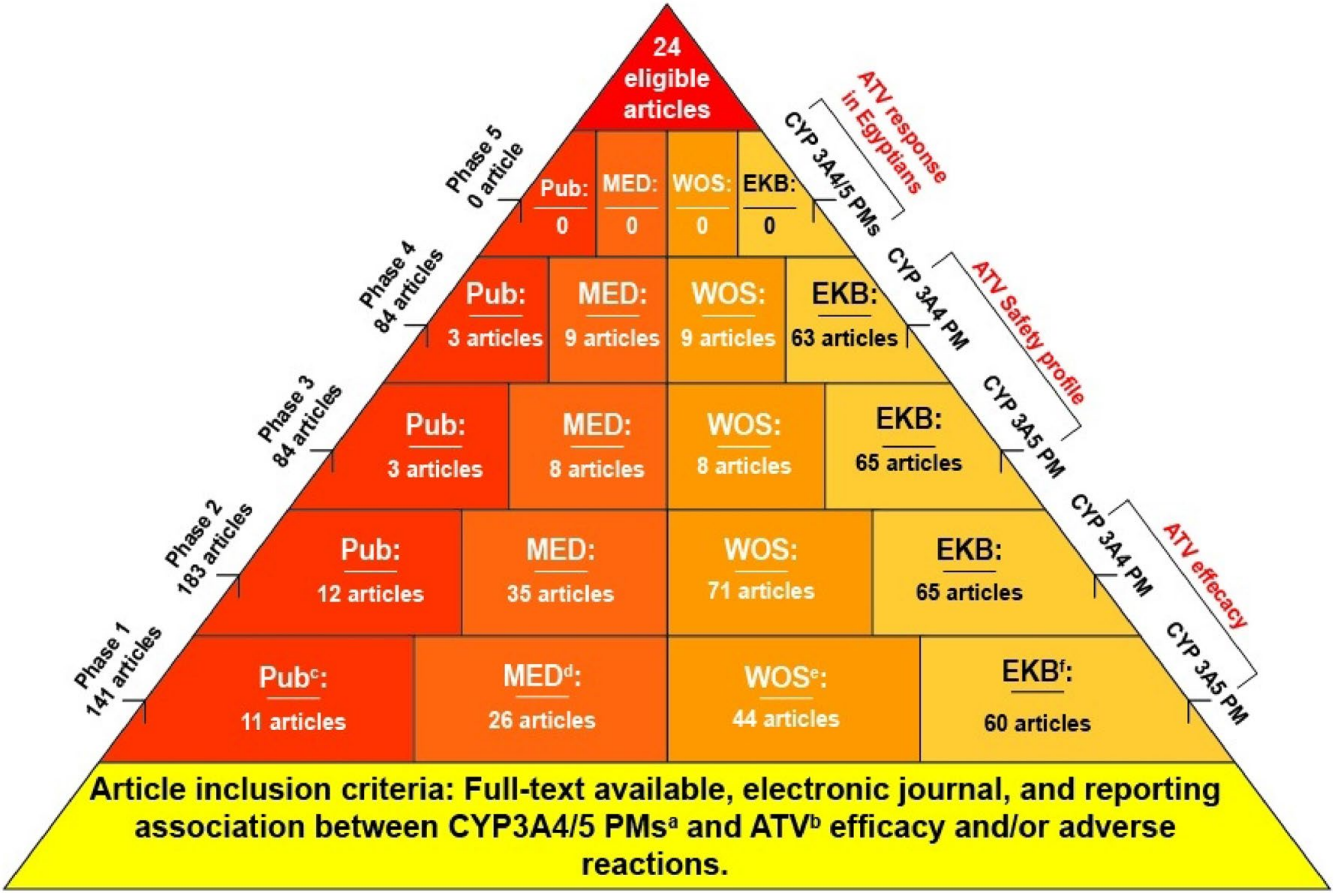
Illustrates the inclusion criteria for selected articles in this review. Five search phases are shown up to March 30, 2022, that involved electronic medical databases like PubMed, Web of Science, MEDLINE, and Egyptian Knowledge Bank (EKB). The following queries were used to get eligible articles for this review: ((CYP3A5) AND (polymorphism)) AND (atorvastatin) for phase 1, ((CYP3A4) AND (polymorphism)) AND (atorvastatin) for phase 2, (((CYP3A5) AND (polymorphism)) AND (atorvastatin)) AND (adverse effect) for phase 3, (((CYP3A4) AND (polymorphism)) AND (atorvastatin)) AND (adverse effect) for phase 4 as well as, ((Cytochromes P450) AND (atorvastatin)) AND (Egypt) for phase 5. This review includes only 24 articles after excluding non-English language literature, books, and literature unrelated to the aim.. a. (PMs): polymorphisms, b.(ATV): atorvastatin, c.(Pub): PubMed, d. (MED): MEDLINE, e. (WOS): Web of Science, f. (EKB): Egyptian Knowledge Bank

Figure 1 shows that an initial list of 492 retrieved articles included 141 articles for phase 1, 183 articles for phase 2, 84 articles for phase 3, 84 articles for phase 4, and no articles for phase 5. Regarding phase 1, the 141 articles included 11 articles from PubMed, 26 articles from MEDLINE, 44 from Web of Science, and 60 from EKB. For phase 2, the 183 articles included 12 articles from PubMed, 35 from MEDLINE, 71 from Web of Science, and 65 from EKB. For phase 3, the 84 articles included three articles from PubMed, eight from MEDLINE, eight from Web of Science, and 65 from EKB. Finally, for phase 4, the 84 articles included three articles from PubMed, nine from MEDLINE, nine from Web of Science, and 63 from EKB. Table 1 illustrates eligible and selected articles (i.e., *n* = 24) included in this review according to the inclusion criteria.

**Table 1:** Main objectives of the 24 articles eligible for inclusion in this review

S.no	Author (s)	Main Objective (s)
1	Dagli-Hernandez et al. [15]	To review clinical trials on pharmacogenomics of statins regarding the Brazilian population
2	Rosales et al. [21]	To evaluate the response to atorvastatin in Chilean hypercholesterolemic patients with PMs^a^ in ABCB1^b^, CYP3A5^c^, and CYP3A4^c^ genes
3	Shitara, Sugiyama. [24]	To review statins' PK^d^ and physicochemical issues and specific aspects such as PMs^a^ that could affect PK^d^
4	Kivistö et al. [25]	To investigate whether CYP3A5^c^ expression results in poor response to statin therapy in Caucasians
5	Zubiaur et al. [26]	To evaluate the impact of SLCO1B1^e^ phenotype on atorvastatin exposure by conducting a candidate gene pharmacogenetic research
6	He et al. [27]	To assess the influence of CYP3A4*1G^c^ variant on atorvastatin PK^d^ in China's Han subjects with CAD^f^
7	Park et al. [28]	To evaluate the contributions of CYP3A5^c^ and CYP3A4^c^ to atorvastatin metabolism
8	Maekawa et al. [29]	To assess, in vitro, the influence of CYP3A4*18^c^ and CYP3A4*16^c^ on the enzymatic function required for the metabolism of several drugs, including atorvastatin
9	Jani et al. [30]	To investigate CYP450^c^ genetic PMs^a^ among Gujarat subjects in India depending on atorvastatin as a probe
10	Poduri et al. [31]	To examine the influence of PMs^a^ of six specific genes on the therapeutic effect of statins in subjects suffering CAD^f^
11	Gao et al. [32]	To investigate the influence of CYP3A4*1G^c^ PM^a^ on statins therapy
12	Peng et al. [33]	To illustrate the association between CYP450^c^ genetic PMs^a^ and response to atorvastatin in Chinese patients with ischemic stroke
13	Kajinami et al. [34]	To study the effect of three CYP3A4^c^ variant alleles on atorvastatin treatment
14	Willrich et al. [35]	To summarize findings from previous studies on variations in responses to statins due to CYP3A^c^ PMs^a^
15	Kadam et al. [36]	To screen LDL-C^g^ level after atorvastatin treatment in Indian carriers of genetic PMs^a^ in several enzymes involved in the pharmacodynamics and PK^d^ of statins
16	Klein et al. [37]	To examine the effect of genetic mutations on the phenotype of CYP3A4^c^ in human hepatocytes and participants using atorvastatin
17	Kolovou et al. [38]	To investigate the effect of CYP3A5*3^c^ PMs^a^ on the lipid profile after atorvastatin or simvastatin treatment
18	Willrich et al. [39]	To assess the impact of CYP3A5^c^ PMs^a^ on statins efficacy in 139 hypercholesterolemic Brazilians
19	Vrablik et al. [40]	To review literature about statin-induced myopathy
20	Becker et al. [41]	To investigate the influence of CYP3A4^c^ and ABCB1^b^ PMs^a^ on intolerance to atorvastatin or simvastatin treatment
21	Xia et al. [42]	To develop and validate a UHPLC-MS/MS^h^ approach for studying atorvastatin calcium PK^d^ in healthy carriers of certain genotypes
22	Liu et al. [43]	To explore the impact of microRNA on the inherited malfunctioning CYP3A4/5^c^ enzymes and atorvastatin metabolism
23	Wilke et al. [44]	To investigate the assumption that carriers of CYP3A5*3^c^ or CYP3A4*1B^c^ are at risk of myopathy due to atorvastatin
24	Benes et al. [45]	To review the risks for ADRs^i^ of commonly recommended statins

a. (PMs): polymorphisms, b. (ABCB1): ATP-binding cassette transporter B1, c. (CYP): Cytochrome P450 enzyme, d. (PK): pharmacokinetics, e. (SLCO1B1): solute carrier organic anion transporter family member 1B1, f.(CAD): coronary artery disease, g.(LDL-C): low-density lipoprotein-cholesterol, h. (UHPLC-MS/MS): ultra-high-performance liquid chromatography coupled with tandem triple quaternary mass spectrometry, i. (ADRs): adverse drug reactions

### Response to atorvastatin

Table 2 demonstrates the effect of CYP3A4/5 genetic variations on atorvastatin response.

**Table 2:** Overview of CYP3A4/5 polymorphisms effect on the response to atorvastatin

Response	Gene	SNP		rs Number	Association
Efficacy	CYP3A4^a^	*1B		*rs2740574*	Decreased TC^c^ and LDL-C^d^ with a significantly elevated HDL-C^b^ in Chilean patients (*P* < 0.001) [21]
					Increased LDL-C^d^ level in American subjects (*P* < 0.05) [24, 34, 35]
					Low LDL-C^d^ serum level reductions in Indians (*P* < 0.05) [36]
		*1G		*rs2242480*	Decreased serum TC^c^ level in Chinese patients (*P* < 0.01) [32]
					Reduced serum LDL-C^d^ level among Chinese patients (*P* = 0.049) [33]
					Low (AUC_0–∞_)^e^ for both atorvastatin and 2-OH-atorvastatin in China’s Han subjects (*P* < 0.05) [27]
		*3 (M445T)		*rs4986910*	Low pretreatment serum LDL-C^d^ level in Americans (*P* = 0.032) [24, 34]
					High LDL-C^d^ lowering response to atorvastatin in Americans; however, it failed to reach statistical significance [24, 34]
		*16 (*T185S*)		*rs12721627*	More than 60% reduced functional activity for atorvastatin *(*in vitro study). Further research is necessary to investigate the clinical relevance [29]
		*17 (189F/S)		*rs4987161*	Increased HDL-C^b^ level after atorvastatin therapy in Indians (*P* < 0.05) [31]
		*22		*rs35599367*	Reduced 2-OH-atorvastatin/atorvastatin (AUC_0–∞_)^e^ ratio in Finnish subjects (*P* < 0.001) [37]
	CYP3A5^a^	*3		*rs776746*	Low serum TC^c^ and LDL-C^d^ levels in Europeans (Caucasian subjects) (*P* < 0.05) [24, 25, 35]
					Decreased LDL-C^d^, TC^c^, and TG^f^ serum levels in Greek subjects (*P* < 0.05) [38]
		*3A	*1D C31611T	*rs17161788*	A slight decline in serum TC^c^ and LDL-C^d^ in non-Afro-Brazilians (*P* < 0.05) [35, 39]
			*3C A6986G	*rs776746*	
Safety	CYP3A4^a^	*1B		*rs2740574*	A significant elevation in atorvastatin C_max_^g^ in Chinese subjects (risk of atorvastatin intolerance due to high atorvastatin exposure) [42]
					Decreased risk of statin intolerance in Dutch subjects, particularly in females and carriers of (3435T) allele of the transporter ABCB1^h^ (*P* < 0.05) [41]
		*1G		*rs2242480*	Increased atorvastatin C_max_^g^ in Chinese volunteers [42]
	CYP3A5^a^	*3		*rs776746*	Severe muscle damage due to the decrease in atorvastatin metabolism in Caucasian (American) subjects with European ancestry (*P* < 0.05) [43, 44]
					Risky elevated atorvastatin C_max_^g^ in Chinese subjects [42]
					Less atorvastatin (acid form) exposure (less AUC ^i^/DW^j^ and C_max_^g^/DW^j^) than CYP3A5*1^a^ (*p* = 0.004 and 0.018, respectively) in Caucasians, Latin Americans, Blacks, and Arabs recruited in a Spanish study [26]

a. (CYP): Cytochrome P450 enzyme, b. (HDL-C): high-density lipoprotein cholesterol, c. (TC): total cholesterol, d. (LDL-C): low-density lipoprotein-cholesterol, e. (AUC0–∞): area under the plasma concentration–time curve, f. (TG): triglyceride, g. (Cmax): maximum plasma concentration, h. (ABCB1): ATP-binding cassette transporter B1, i. (AUC): the area under the curve, j. (DW): dose/weight

#### Atorvastatin therapeutic effect

Atorvastatin is predominantly metabolized to active metabolites by CYP3A4 [21, 24–28]. Moreover, the in vitro study carried out by Park et al. proved that CYP3A4 and CYP3A5 were responsible for 85% and 15% of atorvastatin metabolism, respectively. Moreover, inter-personal variations in CYP3A metabolic pathways are pronounced (20–40-fold), potentially related to genetic polymorphisms of genes encoding CYP3A4/CYP3A5 enzymes. Thus, these genetic variations, particularly CYP3A4 polymorphisms, could substantially impact the therapeutic effect of atorvastatin [28].

##### Effects of CYP3A4 polymorphism

A review article showed that atorvastatin and several statins' metabolic pathways depend on CYP3A4. Therefore, any mutation in this gene could result in a significant alteration in the PK of these statins [24]. The outcome of Maekawa et al. in vitro study illustrated that CYP3A4*16 variant found in East Asia had more than 60% decreased functional activity for atorvastatin. Furthermore, the study concluded that the clinical significance of the results should be examined in other prospective studies [29]. In this context, Jani et al. reported that a genetic mutation in CYP3A4 can result in variation in the pharmacologic properties of statins like atorvastatin. The study found that genetic variations in CYP3A4 gene influenced CYP3A4 enzyme activity and affected atorvastatin metabolism in 125 Indian subjects in Gujarat [30].

**Positive consequences of polymorphism**

Increase in serum HDL-C level: Genetic mutations in CYP3A4 and other five genes affected the therapeutic efficacy of atorvastatin in Indians, suffering from coronary artery disease. Poduri et al. reported CYP3A4 allele (189F/S) was linked to elevated high-density lipoprotein cholesterol (HDL-C) levels after atorvastatin therapy (*P*<0.05) [31].

Decrease in serum total cholesterol: Moreover, Gao et al. found that the decline of total cholesterol (TC) serum level was related to CYP3A4*1G mutation in Chinese subjects with hyperlipidemia. The mean percentage decrease in serum TC level was 20.9±5.0% (*1G/*1G), 17.8±3.8% (*1/*1G), and 16.8±3.3% (*1/*1), respectively (P<0.01). Therefore, this genetic polymorphism in CYP3A4 increased the efficacy of atorvastatin therapy [32].

Decrease in serum LDL-C level: Peng et al. reported that a single nucleotide polymorphism (SNP) of CYP3A4, *rs2242480*, was associated with a reduction in serum LDL-C level among Chinese patients with ischemic stroke (*P* = 0.049) [33]. In this context, Kajinami et al. reported that M445T variant of CYP3A4 gene was associated with a significantly lower pretreatment serum LDL-C levels (11.2%) in carriers of this variant relative to non-carriers. In addition, in the M445T allele carriers, a higher LDL-C lowering response to atorvastatin was detected among 340 Americans suffering from primary hypercholesterolemia, although it failed to reach statistical significance [24, 34].

Improvement in serum lipid and lipoprotein levels: Furthermore, Alexy Rosales, et al. studied Chilean patients and revealed that CYP3A4*1B (-290A>G, *rs2740574*) was linked to enhanced therapeutic outcomes after four weeks of atorvastatin pharmacological treatment. This variant led to a substantial reduction in TC and LDL-C with a significant increase in HDL-C. The mean percentage change in serum TC was − 16.1±9.1% (A/A), and − 24.4±11.8% (A/G), respectively (*P*<0.001). For serum LDL-C, it was − 22.2±13.5% (A/A), and − 36.4±17.8% (A/G), respectively (*P*<0.001). In addition, for serum HDL-C, it was 14.9±13.0% (A/A), and 31.8±16.1% (A/G), respectively (*P*<0.001) [21].

**Negative consequences of polymorphism**

Increase in serum LDL-C level: From the same perspective, CYP3A4 genetic polymorphisms could decrease the pharmacological effect of atorvastatin. For example, Kajinami et al. showed that after 52 weeks of atorvastatin therapy (10 mg/day), the A-290G mutant allele of CYP3A4 (CYP3A4*1B) was related to increased levels of LDL-C in 340 American subjects with hypercholesterolemia (*P*<0.05) [24, 34, 35].

Less serum LDL-C level decline: After 8 weeks of atorvastatin treatment (10 mg/day) among 177 Indians, Kadam et al. illustrated that the variant-allele of CYP3A4 *rs2740574* was associated with a lower LDL-C serum level reductions than the wild-type allele (*P*<0.05) [36].

Decrease in AUC_0–∞_: Klein et al. studied 56 Finnish subjects and analyzed atorvastatin and its dominant metabolite 2-OH-atorvastatin (CYP3A4-dependent) concerning specific SNPs. The results demonstrated that CYP3A4*22, the T variant of the SNP *rs35599367*, was associated with a decrease in 2-OH-atorvastatin/atorvastatin area under the plasma concentration-time curve (AUC0–∞) ratio (*P*<0.001). This study concluded that SNP *rs35599367* (CYP3A4*22) could imply variation in response to atorvastatin and other CYP3A4 substrates [37]. Moreover, He et al. illustrated that the CYP3A4*1G variant affects atorvastatin and 2-OH-atorvastatin PK in 20 Han Chinese subjects with coronary artery disease. CYP3A4*1G/*1G genotype was associated with less AUC0–∞ for both atorvastatin and 2-OH-atorvastatin than *1/*1 or the *1/*1G genotypes (P<0.05) [27].

##### Effects of CYP3A5 polymorphism

Carriers of at least a copy of CYP3A5*1 allele (wild-type) express CYP3A5 protein, whereas CYP3A5*3 homozygotes are designated as CYP3A5 non-expressors [25]. A study reported the genotype frequency of the CYP3A5 genetic variations in 350 unrelated Greek Caucasian cases with primary hypercholesterolemia: 13.4% for expressors and 86.6% for non-expressors (homozygous) subjects [38]. CYP3A5 enzyme is not expressed in about 90% of Caucasians [25].

**Positive consequences of polymorphism**

Improvement in serum lipid and lipoprotein levels: CYP3A5*3 allele led to an improvement in response to atorvastatin. Kari T. Kivistö et al. studied 46 Europeans (Caucasian subjects) and revealed that atorvastatin was significantly less effective in the carriers of CYP3A5*1 (expressors) than in the carriers of CYP3A5*3 (non-expressors). After 12 months of treatment, the mean serum TC and LDL-C levels were higher in the expressors of CYP3A5 (*P*<0.05) [24, 25, 35]. Moreover, Genovefa Kolovou et al. revealed that LDL-C, TC, and triglyceride (TG) serum levels were decreased significantly after atorvastatin treatment in 175 Greek carriers of both CYP3A5*3/*3 and CYP3A5*1/*3 genotypes (*P*<0.05) [38].

**Negative consequences of polymorphism**

Decline in response to atorvastatin: A review by Dagli‑Hernandez et al. about clinical trials in Brazilians showed that variations in the CYP3A5 gene were associated with a reduction in response to atorvastatin and simvastatin therapy [15]. In the same context, a Brazilian study involving 139 subjects illustrated that the CYP3A5*3A variant (*1D and *3C combined variants) was linked to a decrease in cholesterol-lowering response to atorvastatin-4 weeks therapy in non-African subjects only. In this study, 93 subjects with non-African ancestry were recruited, and the remaining 46 individuals were Africans [35, 39]. Non-Africans carrying the CYP3A5*3C allele (*3C/*3C genotype) had less decline in serum TC and LDL-C than carriers of CYP3A5*1A allele after atorvastatin treatment (*P*<0.05). In addition, the CYP3A5*1D analysis illustrated that for the *1D variant (*1D/*1D genotype) among non-Africans, carriers had less reduction of TC and LDL-C levels than carriers of CYP3A5*1A allele after atorvastatin treatment (*P*<0.05) [39].

#### Atorvastatin safety profile

##### Effects of CYP3A4 polymorphism

A review by Vrablik et al. indicated that mutant alleles within CYP3A4 have been proposed to be linked to myopathy as an adverse effect of stains. Moreover, CYP3A4 has been indirectly associated with myopathy in atorvastatin users due to its overrepresentation in these patients needing dose reduction or switching from atorvastatin to alternatives [40, 41]. Furthermore, Xia et al. screened 187 Chinese subjects for wild alleles CYP3A4*1B (*rs2740574*) and CYP3A4*1G (*rs2242480*) in addition to other genes involved in atorvastatin metabolism and transport in vivo. However, only six candidates were enrolled to study atorvastatin PK. As a result, atorvastatin's maximum plasma concentration (Cmax) was significantly elevated (high atorvastatin exposure). In addition, one subject of the candidates was terminated during the study due to atorvastatin intolerance [42]. However, according to Becker et al. cohort study, the G allele of CYP3A4*1B in Dutch users of atorvastatin or simvastatin was linked to a reduced risk for statin intolerance, specifically among females and carriers of the mutant allele (3435T) of the transporter ABCB1 [41].

##### Effects of CYP3A5 polymorphism

Liu et al. in vitro study showed that atorvastatin metabolism is decreased and significantly associated with CYP3A5*3. In addition, the two atorvastatin metabolites, para-OH-atorvastatin and ortho-OH-atorvastatin, were significantly reduced in carriers of CYP3A5*3/*3 [43]. This finding was consistent with severe muscle damage associated with CYP3A5*3/*3 [43, 44]. Wilke et al. conducted an American retrospective case-control study on 137 Caucasian subjects with European ancestry. It showed that the CYP3A5*3 variant was allied to the increase in the serum level of creatine kinase (CK) in the case of individuals with myalgia. The study concluded that individuals with myalgia while using atorvastatin were at a high risk of developing a severe myopathy if they were carriers of the CYP3A5*3/*3 genotype (*P*<0.05) [44, 45]. Also, the previously mentioned Chinese study on atorvastatin PK by Xia et al. illustrated the significant elevation in atorvastatin Cmax. This risky elevation was also associated with the allele CYP3A5*3(*rs776746*) in addition to the previously stated genes for atorvastatin metabolism and other two genes for atorvastatin transport: solute carrier organic anion (SLCO) transporter; (SLCO1B1 388A>G (*rs2306283*) and SLCO1B1 521T>C(*rs4149056*)) [42]. Quite the opposite, a Spanish pharmacogenetic study by Zubiaur et al. on 156 subjects (81 Caucasians, 70 Latin Americans, and 5 Blacks or Arabs) showed that atorvastatin accumulation was higher among CYP3A5*1/*1 carriers than *1/*3 or *3/*3 carriers. The authors justified this novel finding by illustrating the first-pass effect on the administered acid form of atorvastatin. Therefore, regarding the CYP3A5*1/*1 genotype, atorvastatin was metabolized to a greater extent in the gut. This genotype resulted in active metabolites and inhibitors of CYP3A4 (responsible mainly for atorvastatin metabolism). Consequently, CYP3A5*3 variant was significantly linked to lower atorvastatin Cmax than CYP3A5*1 (p = 0.018) [26].

## Discussion

The association studies of CYP3A4 and CYP3A5 polymorphisms with response to atorvastatin treatment were inconsistent [21, 23].

### Atorvastatin therapeutic effect

#### Effects of CYP3A4 polymorphism

As mentioned in this review, Rosales et al. researched Chilean subjects and showed that CYP3A4*1B was associated with better therapeutic outcomes after atorvastatin therapy [21]. However, the same variant (CYP3A4*1B) was linked to high levels of LDL-C in Americans after atorvastatin treatment, as reported by Kajinami et al. [24, 34, 35]. On the other hand, research on the Indian population has shown no significant relation between the CYP3A4*1B gene (*rs2740574*) and low values of LDL-C as a response to atorvastatin therapy [31, 46]. Reports for CYP3A4*1B metabolic activity were conflicting [34]. Rosales et al. attributed the positive therapeutic consequences after atorvastatin treatment to CYP3A4 metabolic lower activity in vivo due to the CYP3A4*1B allele. However, the study was restricted by the sample size [21]. On the contrary, Kajinami et al. found serum LDL-C elevation was consistent with CYP3A4-increased enzyme activity [34].

#### Effects of CYP3A5 polymorphism

In Greek subjects, Kolovou et al. showed that although HDL-C levels did not vary meaningfully after atorvastatin treatment, the LDL-C, TC, and triglyceride (TG) serum levels were decreased significantly in both genotypes CYP3A5*3/*3 and CYP3A5*1/*3 [38]. In addition, Kivistö et al., while researching European Caucasian subjects, revealed that the CYP3A5*3 variant led to an enhanced response to atorvastatin [24, 25, 35]. However, the Brazilian study showed that the CYP3A5*3A variant was associated with a decreased cholesterol-lowering response to atorvastatin in subjects with non-African ancestry [35, 39].

In contrast, research on Chilean patients showed that both the G and A alleles of the CYP3A5*3 (rs776746) variant did not affect response to atorvastatin treatment [21]. In addition, a retrospective cohort study performed among Caucasian subjects illustrated no association between the CYP3A5*3 allele and atorvastatin PK [23, 47]. Differences among the mentioned studies indicate that the relationship between the CYP3A5*3 allele and response to atorvastatin could depend on factors such as the period of treatment and ethnicity [39].

### Atorvastatin safety profile

#### Effects of CYP3A4 polymorphism

In Chinese subjects, Xia et al. showed that CYP3A4*1B(*rs2740574*) and other genes involved in atorvastatin metabolism and transport in vivo were associated with risky high atorvastatin exposure [42]. However, in Dutch users of atorvastatin or simvastatin, according to the research of Becker et al. the same variant CYP3A4*1B was associated with a low risk for statin intolerance [41].

Conversely, an open-label randomized study illustrated that CY3A4*1B (*rs2740574*) was not associated with atorvastatin therapy intolerance, the elevation of CK, or muscle pain in Caucasians as well as African American subjects [23, 48].

#### Effects of CYP3A5 polymorphism

Regarding Caucasian subjects, Wilke et al. concluded that patients with myalgia while using atorvastatin were at significant risk of suffering severe muscle damage if they were carriers of CYP3A5*3/*3 [44, 45]. However, Zubiaur et al. reported that high risky atorvastatin exposure was associated with CYP3A5*1/*1. Noticeably, more research is required regarding the controversy with this novel finding [26].

In opposition, a study involving Han Chinese patients demonstrated that the G allele of the CYP3A5*3 (*rs776746*) variant was not associated with myotoxicity as an adverse effect of atorvastatin therapy [49]. Furthermore, the same result was demonstrated in a case-control study in which indigenous American, sub-Saharan, East Asian, and European subjects were recruited [23, 50].

### CYP3A4/5 polymorphisms among Egyptian population

As far as we can report for the Egyptians, the effect of CYP3A4/5 genetic polymorphisms on the response to atorvastatin treatment was understudied. However, some pharmacogenetic studies on Egyptians (Table 3) were performed to better define “Egyptian” genetic variants and assess their significance for common disease states or drugs other than the one under investigation [51].

**Table 3:** Main objectives of the articles that investigated CYP3A4/5 polymorphisms among the Egyptian population

Sl. no.	Author (s)	Main Objective (s)
1	Mutawi et al. [52]	To examine the main allelic PMs^a^ of CYP3A4^b^, CYP3A5^b^, and CYP2D6^b^ in the Egyptian population
2	Sharaki et al. [53]	To study the effect of CYP3A4^b^ rs4646437C > T and MDR1^c^ G2677T/A genetic variations on cyclosporine dosing in renal transplant Egyptian recipients
3	Abd El Wahab et al. [54]	To identify the frequency of CYP1A1*2C^b^ and CYP3A5*3^b^ genetic polymorphisms in Egyptians suffering acute myeloid leukemia and assess their contribution to the development of leukemia in Egyptians
4	Mendrinou et al. [55]	To determine CYP3A5*3^b^ allelic frequency in Egyptian renal transplanted patients and evaluate the effect of this genetic variant on tacrolimus dose requirements
5	Bedewy, El-Maghraby [56]	To identify the frequencies of CYP3A5*3^b^ and SLCO1B3^d^ (T334G) in chronic myeloid leukemia cases receiving imatinib therapy and assess the impact of these polymorphisms on the response to imatinib
6	Abo El Fotoh, et al. [57]	To determine the effect of CYP3A5*3^b^ and SCN1A^e^ c.3184 A/G polymorphisms on pharmaco-resistance in Egyptian epileptic pediatric patients

a. (PMs): polymorphisms, b. (CYP): Cytochrome P450 enzyme, c. (MDR1): multidrug resistance protein 1, d. (SLCO1B3): solute carrier organic anion transporter family member 1B3, e. (SCN1A): sodium voltage-gated channel alpha subunit 1

Regarding CYP3A4 genetic variations among Egyptians, pharmacogenomic research performed at the pediatric hospital, Faculty of Medicine, Mansoura University has reported an allele frequency of 2% for the CYP3A4*22 allele. It is the first study illustrating the CYP3A4*22 variant frequency in the Egyptian population [52]. Moreover, the frequency of an additional variant *rs4646437* in the CYP3A4 gene was reported in another Egyptian study to be 20%. The study involved 50 Egyptian patients after kidney transplantation at the Renal Transplantation Unit of Alexandria University Hospital [53].

Concerning CYP3A5*3 polymorphism among Egyptians, a study was conducted at the adult oncology department, National Cancer Institute, Cairo University. The results showed that the frequency of CYP3A5*3 was higher at 81.5% in acute myeloid leukemia cases compared to controls [54]. Another Egyptian study at Urology and Nephrology Center, Mansoura University Hospital, published in August 2020, demonstrated that the CYP3A5*3 allele was the most prevalent variant (85.53%) in 76 renal transplantation recipients. This study was the first Egyptian research focusing on the personalization of tacrolimus doses in Egyptian cases according to the CYP3A5 genotypes [55]. Another research on Egyptian cases of chronic myeloid leukemia (CML) was performed at the Hematology Department, Medical Research Institute, Alexandria University. This research was designed to illustrate the role of CYP3A5*3 polymorphism in determining the response to imatinib in 86 CML Egyptian cases. The results showed that the frequency of CYP3A5*3 was 53% and 69% in 78 (early chronic phase) cases and eight (accelerated phase) cases, respectively [56]. Moreover, another Egyptian pharmacogenetic research on 130 epileptic children was held at Pediatrics Department, Menoufia University Hospital. The research aimed to show the role of CYP3A5*3 genetic variation in predicting resistance to antiepileptic medications in Egyptian epileptic pediatric cases. The prevalence of CYP3A5*3 was 76.9% and 77.7% in epileptic (*n*=130) and control (*n*=65) participants, respectively [57]. Due to the limited data about pharmacogenes of significant clinical importance, 145 healthy unrelated Egyptian children were included in research at Mansoura University Children’s Hospital. This pharmacogenomic research aimed to screen common genetic variants in certain CYPs, including CYP3A5, among the Egyptian population. The prevalence of variant 6986A>G (*rs776746*) in the gene CYP3A5 was 86.2% [52].

Findings of association studies of CYP3A4/5 genetic polymorphisms with response to atorvastatin treatment varied among different ethnicities. CYP3A4*1B (*rs2740574*) and CYP3A5*3 (*rs776746*) were reported in the literature as either associated with the risk for atorvastatin intolerance in a particular population or associated with a low possibility for risky atorvastatin exposure in another population. In addition, both variants were reported to be either associated with better therapeutic outcomes after atorvastatin treatment in a definite population or with poor therapeutic consequences in another. Focusing on the Egyptian population, both alleles C and T of the variant CYP3A4*1B (*rs2740574*) in the CYP3A4 gene, as far as we know, were not studied before. In addition, the effects of genetic polymorphisms of both CYP3A4 (*rs2740574* C/T) and CYP3A5*3 (*rs776746* T/C) on atorvastatin PK or its induced adverse effects were not previously studied in Egyptians. Consequently, prospective pharmacogenomics studies amongst Egyptians will assist many people in this population suffering from hypercholesterolemia and are at high risk of CVD complications. Therefore, the outcomes would tailor atorvastatin treatment based on the patient’s genotype.

## Conclusions

CYP3A4/5 genetic variants have been studied for their potential associations with response to atorvastatin treatment. However, the findings differed according to ethnicity. Therefore, more pharmacogenetic research in various populations worldwide, like the Egyptian population, is required to elucidate the impact of genetic polymorphism of these CYP enzymes on atorvastatin response and risk of side effects with genotype-guided dosing and precision medicine initiatives.

## Data Availability

The authors confirm that all relevant data are included in the article.
